# Anthracycline Cardiotoxicity: Prevalence, Pathogenesis and Treatment

**DOI:** 10.2174/157340311799960645

**Published:** 2011-11

**Authors:** Maria Volkova, Raymond Russell

**Affiliations:** 1 Section of Cardiovascular Medicine, Yale University School of Medicine; 2 Smilow Cancer Hospital

**Keywords:** Left ventricular dysfunction, doxorubicin, oxidant stress.

## Abstract

Anthracyclines, such as doxorubicin and idarubicin, remain an important class of chemotherapeutic agents. Unfortunately, their efficacy in treating cancer is limited by a cumulative dose-dependent cardiotoxicity, which can cause irreversible heart failure. In this review, we discuss the pathogenesis and incidence of anthracycline-induced cardiotoxicity as well as methods to detect, prevent and treat the condition.

## INTRODUCTION

Cancer chemotherapy has made remarkable advances in the treatment of both solid and hematologic malignancies, allowing in many patients the hope for a cure of their cancer. However, these therapies are not without their complications. In this review, we will address the cardiac toxicity of one of the most common classes of chemotherapeutic agents, the anthracyclines, focusing on the prevalence, diagnosis, treatment and prevention of the cardiotoxicity that should be understood by the cardiologist.

## PREVALENCE OF ANTHRACYCLINE-INDUCED CARDIOTOXICITY

The family of anthracycline drugs originated in the 1950’s with the identification of daunorubicin from the soil bacterium Streptomyces peucetius [1]. In the 1960’s, daunorubicin was found to be quite effective in treating leukemias and lymphomas [2]. Also in the 1960’s, a derivative of daunorubicin, 14-hydroxydaunomycin or Adriamycin (later to be renamed doxorubicin), was identified and shown to be a more effective antitumor agent [3, 4]. Since these initial investigations of this class of antitumor agents, anthracycline chemotherapeutic agents have been employed in the treatment of a wide variety of solid organ tumors and hematologic malignancies, including leukemia, lymphoma, breast cancer, lung cancer, multiple myeloma and sarcoma.

Although doxorubicin has become one of the most effective chemotherapeutic agents, it was noted early on that its use was complicated by the development of heart failure [5, 6]. In a retrospective analysis of over 4000 patients receiving doxorubicin performed by Von Hoff and colleagues, 2.2% of the patients developed clinical signs and symptoms of congestive heart failure [6]. Because the study was based on clinician-identified signs and symptoms of congestive heart failure, decreases in left ventricular function without overt symptoms were not identified and the authors acknowledged that “the incidence of drug-induced subclinical left ventricular dysfunction may have been higher”. This study went on to demonstrate that one of the greatest determinants of the development of heart failure is the cumulative dose of doxorubicin, with a sharp increase in the prevalence of heart failure occurring at a cumulative dose of 550 mg/m^2^. The cumulative dose of anthracycline is a clinical factor that continues through today to predict the development of heart failure. In addition, the use of smaller, divided doses decreased the likelihood of developing heart failure.

Subsequent studies measured changes in left ventricular ejection fraction (LVEF) with anthracycline chemotherapy and confirmed a cumulative dose-dependent decrease in LVEF, particularly at cumulative doses of doxorubicin >350 mg/m^2^ [7, 8]. For the most part, these decreases in left ventricular function were asymptomatic, and if the cardiotoxicity was no more than moderate (a decrease in LVEF of ≥15% to an absolute LVEF between 30 and 45%), stabilization of the LVEF generally occurred with discontinuation of anthracycline therapy.

In a retrospective analysis of three trials of doxorubicin treatment of breast cancer or small cell lung cancer (Multicenter trials 088001, 088006 and 088002) in which LVEF was measured by equilibrium radionuclide angiography (ERNA), Swain *et al.* demonstrated that 5.1% of patients had evidence of congestive heart failure or a significant decline in left ventricular function and confirmed the cumulative dose-dependence of doxorubicin cardiotoxicity [9]. Furthermore, in contrast to previous studies, they demonstrated an increased risk of cardiotoxicity at doses of doxorubicin (≤300 mg/m^2^) that had previously been considered unlikely to cause left ventricular dysfunction (Table **1**). Interestingly, histopathologic changes can be seen in endomyocardial biopsy specimens from patients who have received as little as 240 mg/m^2^ of doxorubicin [10, 11].

This progressive cardiotoxicity usually occurs after the completion of treatment with anthracyclines, and may become apparent within one year of the completion of treatment (early onset chronic cardiotoxicity) or many years after chemotherapy has been completed (late onset chronic cardiotoxicity). This particular aspect of anthracycline-induced cardiotoxicity is particularly relevant in adult survivors of pediatric malignancies [12, 13]. Up to 65% of patients with a history of a childhood malignancy treated with doxorubicin can have echocardiographic evidence of left ventricular contractile abnormalities [14]. In the Childhood Cancer Survivor Study, a study of 14,358 5-year survivors of childhood malignancies, use of <250 mg/m^2^ of anthracycline was associated with a 2.4-fold higher risk of developing congestive heart failure compared to those patients who did not receive anthracyclines [13]. This risk increased to 5.2-fold with the use of ≥250 mg/m^2^ of doxorubicin. In adult patients with breast cancer treated with adjuvant chemotherapy that included anthracyclines, Abu-Khalaf *et al.* demonstrated that the median absolute change in LVEF from baseline was –5.5% seven years after receiving anthracyclines [15]. Furthermore, 12% of their cohort had an LVEF below the lower limit of normal following chemotherapy.

In addition to this late cardiotoxicity, a rare form of acute anthracycline cardiotoxicity has been described in case reports and small patient series [16-18]. The manifestations of this potentially lethal cardiotoxicity may include pericarditis and arrhythmias in addition to left ventricular dysfunction [16, 19]. In contrast to the late cardiotoxicity of anthracyclines, improvement in left ventricular function has been noted to occur in some patients [16, 18]. Furthermore, the mechanism responsible for the acute toxicity may involve an inflammatory response [11], which differs from the generally accepted cause of the chronic anthracycline cardiotoxicity discussed below. A recent case report suggested that treatment with anthracyclines may also produce a stress-induced (takotsubo) cardiomyopathy [20].

## MECHANISMS OF CARDIOTOXICITY

Chemotherapeutic cardiotoxicity can be characterized as either type 1 or type 2 cardiotoxicity based on the effect of the agent on cardiomyocytes [21]. Type I cardiotoxicity is caused by cardiomyocyte death, either through necrosis or apoptosis, and as a result is not reversible. Type II cardiotoxicity is caused by cardiomyocyte dysfunction rather than cell death and therefore may be reversible. The long-term cardiotoxicity caused by the anthracyclines includes cardiomyocyte death and therefore represents a type I toxicity. Understanding the etiology of this cardiotoxicity has allowed the development of preventive strategies to combat the development of permanent cardiac damage.

While the primary mechanisms responsible for the efficacy of doxorubicin in killing rapidly dividing cancer cells are related to DNA damage, the toxicity that doxorubicin exhibits in cardiomyocytes is related to free radical formation caused by doxorubicin metabolism. Specifically, the reduction of doxorubicin by NADH dehydrogenase in mitochondrial respiratory complex I, forms a semiquinone radical that can react with molecular oxygen to form the superoxide radical [22]. Subsequently, redox cycling results in the production of hydrogen peroxide and the hydroxyl radical [23]. In addition, formation of doxorubicin-iron complexes may catalyze a Fenton reaction (Fe^2+^-catalyzed conversion of hydrogen peroxide to hydroxyl radical) resulting in the generation of reactive oxygen species [24, 25]. It is likely that cardiomyocytes are much more sensitive to the oxidant stress caused by doxorubicin because of their high reliance on oxidative substrate metabolism and therefore the high fraction of cardiomyocyte volume made up by mitochondria compared to glycolytic tumor cells. In fact, oxidant damage by doxorubicin in tumor cells is only seen at very high doxorubicin concentrations [26].

Transgenic mouse models have further demonstrated the importance of modulating reactive oxygen species production in ameliorating the cardiotoxic effects of doxorubicin. Overexpression of manganese-dependent superoxide dismutase (Mn-SOD) decreases apoptosis and improves left ventricular function in mice treated with doxorubicin while deletion of Mn-SOD enhances the cardiotoxic effects of the agent [27, 28]. Recent work from our lab has demonstrated that doxorubicin metabolism in the cardiomyocyte involves activation of the transcription factor, aryl hydrocarbon receptor, which increases expression of drug metabolizing proteins [29]. Deletion of aryl hydrocarbon receptor from mice results in increased cardiomyocyte reactive oxygen species generation and apoptosis in response to doxorubicin treatment as well as increased left ventricular dysfunction [29]. Furthermore, deletion of proteins that can stimulate the production of free radicals from doxorubicin (e.g., nitric oxide synthase 3) can prevent the decline in left ventricular function following doxorubicin treatment [30].

**Fig. (1):**
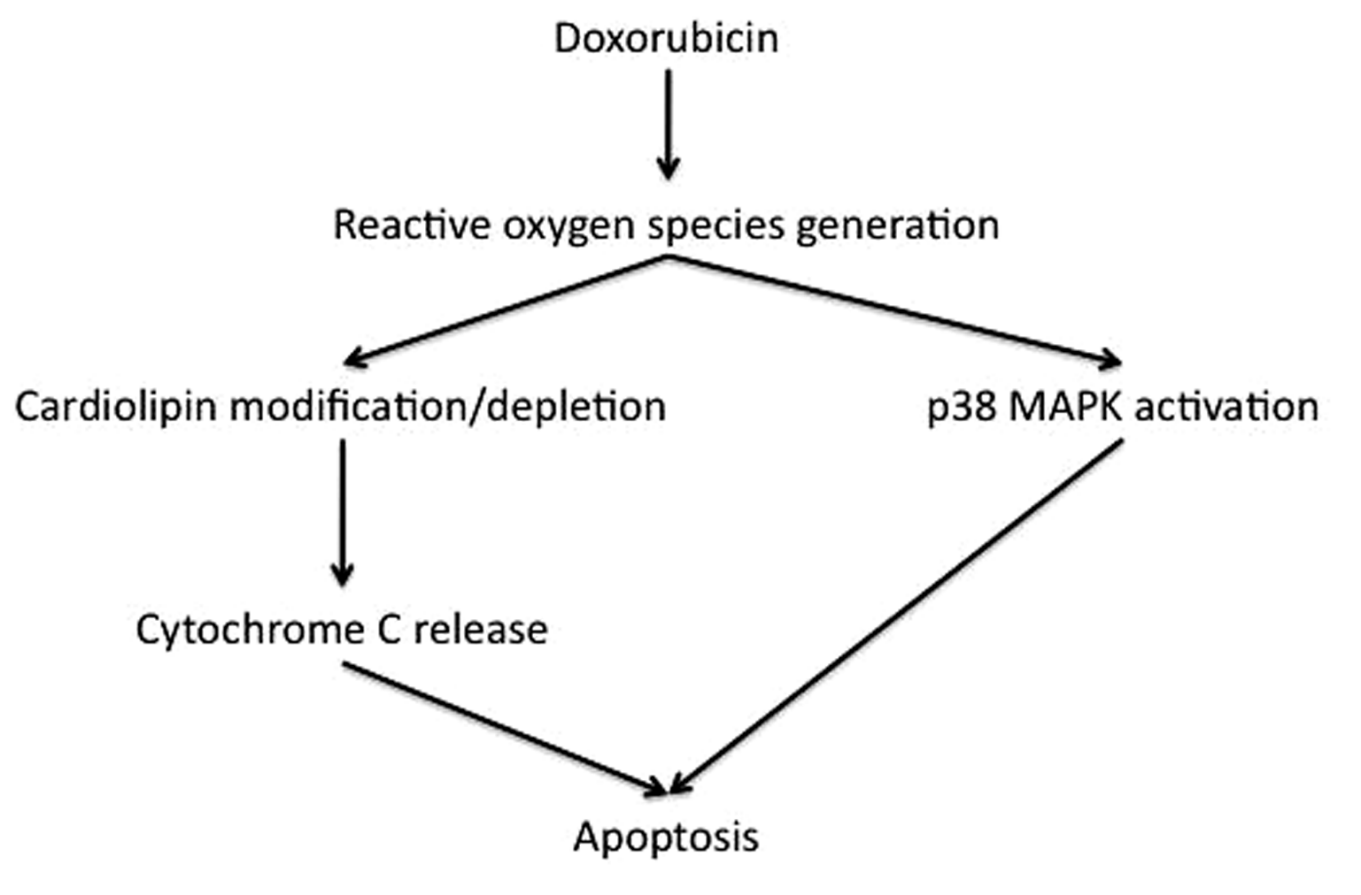
Cellular mechanisms responsible for increased apoptosis in cardiac myocytes by doxorubicin.

The reactive oxygen species that are produced by doxorubicin metabolism in cardiomyocytes subsequently cause cell death through apoptotic pathways (Fig. **1**) [31-33]. Specifically, doxorubicin treatment of cardiomyocytes causes caspase 9 and caspase 3 activation [34-36], opening of the mitochondrial permeability transition pore and subsequent release of cytochrome C into the cytosol [37, 38]. Furthermore, doxorubicin binds directly to the mitochondrial phospholipid, cardiolipin, disrupting the association of inner mitochondrial membrane proteins with cardiolipin [39-41], which could enhance cytochrome C release in response to oxidant stress.

The activation of apoptosis by doxorubicin is mediated in part by p38 MAPK activation [42, 43]. Although there is evidence that early after doxorubicin exposure, there is initial upregulation of the expression of the antiapoptotic proteins, Bcl-XL and Bcl-2 [37] followed by decreases in their expression [44], and increasing the expression of Bcl-XL or Bcl-2 can protect against doxorubicin-induced cardiotoxicity [45-48].

Cardiomyocyte death is determined by the balance between the above cytotoxic pathways and cytoprotective pathways and understanding these cytoprotective pathways may provide insights into novel treatments that may decrease the toxicity of anthracyclines. Antioxidant treatment can cause Akt activation, [49-51] and it has been shown that increasing Akt activity through adenoviral vector delivery of constitutively active Akt improves left ventricular function following treatment with doxorubicin [52]. Furthermore, the protective effects of Akt activation include the inhibition of apoptosis, through the inactivation of caspase 9 and caspase 3 activation [35, 53]. In addition, Akt activation is associated with increased expression of the antiapoptotic protein, Bcl-2 [54, 55]. Of clinical interest, one study has demonstrated that neuregulin-1β, the ligand of the epidermal receptor kinase, erbB2, activates Akt in adult rat ventricular myocytes and decreases myocyte disarray [56]. ErbB2 is the target of the chemotherapeutic agent, trastuzumab, which is thought to inactivate erbB2 receptor signaling in breast cancer cells and has been shown to also cause left ventricular dysfunction [57].

## IDENTIFICATION OF PATIENTS AT RISK FOR ANTHRACYCLINE-INDUCED CARDIOTOXICITY

As noted above, the greatest risk factor for anthracycline-induced cardiotoxicity is the cumulative dose. In addition to the cumulative dose, other risk factors have been identified that increase the risk of anthracycline-induced cardiotoxicity, including extremes of age, female gender, prior mediastinal radiation therapy, hypertension [6], concomitant treatment with cyclophosphamide, trastuzumab or paclitaxel, and the presence of cardiac disease (Table **2**). Of particular interest is the interaction between anthracyclines, such as doxorubicin, and trastuzumab, given the relatively common use of the latter agent for adjuvant therapy for breast cancer. A recent review of two large trials comparing the use of chemotherapy with doxorubicin and cyclophosphamide alone with the use of the two agents in addition to adjuvant trastuzumab demonstrated an incidence of congestive heart failure of 0.45% for chemotherapy alone versus 2.0% for chemotherapy plus adjuvant trastuzumab [58]. While patients with preexisting left ventricular dysfunction are at increased risk of developing anthracycline-induced cardiotoxicity, at least one study suggests that significant decreases in LVEF from the baseline value do not occur for cumulative doses up to 350 mg/m^2^ [59], however, frequent monitoring of left ventricular function is essential.

Because anthracycline-induced cardiotoxicity is a type I cardiotoxicity, detection of cardiomyocyte injury would be expected to identify those patients who may go on to develop left ventricular dysfunction. While several biomarkers have been investigated, troponin I has the greatest evidence of detecting changes in left ventricular function early. Studies by Cardinale *et al.* demonstrated that patients receiving high-dose doxorubicin therapy who had an increase in the plasma troponin I ≥0.5 ng/ml 12-72 hours after treatment with doxorubicin had a greater decrease in LVEF after 7 months [60, 61]. In addition, individuals who have a persistent increase in troponin I following treatment with anthracyclines have a greater decrease in LVEF compared to those who only have an early troponin I release [62].

It is reasonable to assume that there may be a genetic predisposition to developing anthracycline-induced cardiotoxicity in some patients and there is a great deal of interest in identifying gene polymorphisms that are associated with a greater sensitivity to the cardiotoxic effects of anthracyclines. In a study of patients with non-Hodgkin’s lymphoma, Wojnowski *et al.* evaluated single-nucleotide polymorphisms in 82 candidate genes that they hypothesized may be associated with the development of anthracycline cardiotoxicity [63]. They identified polymorphisms in genes encoding three proteins: NAPD(H) oxidase, which is responsible for reactive oxygen species generation, and the doxorubicin efflux transporters MRP1and MRP2. While these studies focused on a specific group of genes, there have been no genome-wide association studies to determine other genes that may identify individuals at increased risk of developing anthracycline cardiotoxicity.

## PREVENTION AND TREATMENT OF ANTHRACYCLINE-INDUCED CARDIOTOXICITY

Anthracycline-induced cardiotoxicity is due in large part to the generation of free radicals from doxorubicin through mitochondrial redox cycling of doxorubicin in the cardiomyocyte, which ultimately results in left ventricular dysfunction, and in the most severe cases, congestive heart failure. As a result, efforts aimed at decreasing this exogenous oxidant stress may decrease the cardiotoxicity of doxorubicin and attenuate the left ventricular dysfunction associated with use of this chemotherapeutic agent. Reduction of oxidant stress from circulating anthracyclines can be achieved through a variety of mechanisms. Delivery of doxorubicin in a PEGylated liposomal form decreases the circulating concentrations of free doxorubicin and result in selective uptake of the agent in tumor cells. Use of PEGylated liposomal doxorubicin has been shown to decrease the cardiotoxicity of this anthracycline, even at doses >500 mg/m^2^ [64, 65]. The antioxidant, probucol, has been shown to prevent the decrease in LVEF in animal models of doxorubicin cardiotoxicity [66, 67]. The beta-adrenergic blocker, carvedilol, has also been shown to protect against doxorubicin-induced left ventricular dysfunction through its antioxidant properties [68, 69]. Interestingly, the antioxidant properties of vitamin E have been shown to be ineffective in preventing left ventricular dysfunction in animals treated with doxorubicin [70, 71]. While the above studies, performed in animal models of doxorubicin cardiotoxicity and in *in vitro* experiments, have generally shown the favorable effects of antioxidant therapy, the results of clinical studies of antioxidants have been inconsistent due to differences in the antioxidant agent used, timing of therapy, type of malignancy and chemotherapeutic regimen [72].

As discussed earlier, reactive oxygen species can be generated by the interaction of doxorubicin with non-heme iron through the Fenton reaction. Based on this mechanism of reactive oxygen species generation, the iron chelating agent, dexrazoxane, was introduced as a treatment to prevent anthracycline-induced cardiotoxicity. Dexrazoxane has been shown in multiple trials to reduce the incidence of congestive heart failure and decreases in LVEF [73, 74]. This cardioprotective effect of dexrazoxane is observed even if it is started after patients have received 300 mg/m^2^ of doxorubicin [73]. Because there is some concern that dexrazoxane may attenuate the chemotherapeutic efficacy of doxorubicin [74, 75], it is recommended that its use be initiated only after a patient has received 300 mg/m^2^ of doxorubicin.

Other agents have shown promise in reducing cardiotoxicity, particularly in patients at increased risk of developing cardiotoxicity. As noted above, patients who have an increase in the plasma troponin I during treatment with doxorubicin are at greater risk for chronic anthracycline-induced cardiotoxicity [60-62]. Treatment with enalapril prevented the late decline in LVEF in patients with elevated troponin I values during doxorubicin treatment [76]. In addition, in patients receiving high doses of doxorubicin (>500 mg/m^2^), treatment with carvedilol prevented the decline in LVEF [77]. Therefore, treatment with ACE inhibitors and/or carvedilol should be considered in patients at risk for developing anthracycline cardiotoxicity.

The decision to treat a malignancy using an anthracycline chemotherapeutic agent must be based on the potential benefit of treating the cancer versus the potential cardiac risk. This requires open communication between the oncologist and the cardiologist to establish the merits and risks of anthracycline treatment. In patients that have no risk factors for anthracycline-induced cardiotoxicity Table **2** who will benefit greatly from treatment with anthracyclines, the decision to utilize antracyclines is straightforward, and monitoring of left ventricular function is routine. At the other end of the spectrum, those patients with two or more risk factors for anthracycline-induced cardiotoxicity who are unlikely to benefit from use of anthracycline should not receive the agent. For the remainder of patients, the relative risk and benefits of anthracycline use will determine impact decisions concerning whether more frequent monitoring (e.g., before every cycle of chemotherapy) or the addition of cardioprotective agents such as ACE inhibitor should be considered.

Similar evaluations of the risk and benefit must be made in patients that develop anthracycline-induced cardiotoxicity and it must be decided whether to continue anthracycline therapy. In this scenario, the degree of left ventricular dysfunction can play a significant role in determining whether to continue treating with an anthracycline agent. For example, in patients who may benefit greatly from a chemotherapy regimen that includes doxorubicin, it may be decided to continue that therapy if the LVEF is >40%, provided that cardioprotective treatments are instituted and frequent monitoring of left ventricular function is performed.

In patients who develop left ventricular dysfunction during or after receiving anthracycline-based chemotherapy, it is important to consider other causes of heart failure. In particular, coronary artery disease must be considered in adult patients with risk factors for coronary artery disease and an ischemia workup initiated. In addition, endomyocardial biopsy may be considered in patients in whom there is question as to the cause of left ventricular dysfunction or in helping to determine if anthracycline chemotherapy should be continued, especially if the patient has received high doses of the agent [78].

There are no treatments that are specific to anthracycline-induced congestive heart failure. Rather, treatment should utilize standard therapies for congestive heart failure, including ACE inhibitors, beta-blockers and loop diuretics for volume management. In a recent study of patients with an anthracycline-induced decrease in LVEF ≤45%, treatment with enalapril and carvedilol resulted in normalization of LVEF in 42% of patients [79]. These responders had a higher LVEF after the onset of congestive heart failure compared to partial responders (those whose LVEF increased >10% but did not normalize) and nonresponders and had therapy initiated sooner than partial responders and nonresponders [79]. This is particularly important in light of recent data that indicates that only 31% of patients receiving chemotherapy with an asymptomatic decrease in LVEF receive an ACE inhibitor or angiotensin receptor blocker, 35% receive a beta blocker and 42% are referred for cardiology consultation [80]. This last fact once again emphasizes the importance of communication between the oncologist and cardiologist.

## ASSESSMENT OF LEFT VENTRICULAR FUNCTION

Left ventricular systolic and diastolic function can be assessed with a variety of noninvasive imaging techniques, including equilibrium radionuclide angiography (ERNA), transthoracic echocardiography, cardiac magnetic resonance imaging, and cardiac computed tomography. Of these, ERNA and transthoracic echocardiography are the most commonly used methods in clinical practice to monitor left ventricular function in patients receiving potentially cardiotoxic chemotherapy. ERNA is count-based and therefore quantitative, but does expose the patient to approximately 5 miiliSieverts. Newer gamma cameras that employ more sensitive cadmium-zinc-telluride crystals may allow for dose reductions, thereby reducing radiation exposure 5-fold. In contrast, transthoracic echocardiography does not require radiation exposure, and if careful planimetry of the left ventricular cavity is performed, an accurate quantitative measure of LVEF can be obtained. In addition, echocardiography provides additional anatomic assessment of the myocardium, valvular structure and great vessels that cannot be obtained by ERNA. In patients who do not image well by transthoracic echocardiography, use of echo contrast material or ERNA may be substituted to assess the LVEF. While either ERNA or transthoracic echocardiography may be used to assess left ventricular function in patients receiving chemotherapy, it is essential that the same technique be used in serial examinations to avoid variability between the two methods.

## SUMMARY

Anthracyclines remain an important class of drugs in the treatment of cancer, but also remains a problematic chemotherapeutic agent given their cardiotoxic effects. Close monitoring of patients is essential to decrease the risk of anthracycline-induced cardiotoxicity as is the early implementation of cardioprotective therapies, especially in those individuals at increased risk of developing left ventricular dysfunction in response to anthracyclines. The likelihood of avoiding or reducing the cardiotoxic effects of these agents while improving the oncologic benefits of the therapy can be increased by close collaboration between the oncologist and cardiologist.

## Figures and Tables

**Table 1.** Dose Related Risk of Doxorubicin-Induced Congestive Heart Failure (Based on Data from (9))

Cumulative Dose (mg/m^2^)	Patients with CHF (%)
150	0.2
300	1.6
450	3.3
600	8.7

**Table 2.** Factors Associated with Increased Risk of Anthracycline- Induced Cardiotoxicity

Age >65 years or <4 years
Female gender
Hypertension
Preexisting cardiac disease
Mediastinal radiation
Treatment with cyclophosphamide, paclitaxel, or trastuzumab
Cumulative anthracycline dose
Higher individual anthracycline doses
